# Supplementary material for: Investigating the Formation of Structural Elements in Proteins Using Local Sequence-Dependent Information and a Heuristic Search Algorithm
Source: Molecules. 2019 Mar 22;24(6):1150. doi: 10.3390/molecules24061150 (PMC6471799; doi:10.3390/molecules24061150)
Supplement: Supplementary file 1 [file molecules-24-01150-s001.pdf]

Supplementary material for article:

Investigating the formation of structural elements in proteins using local sequence-dependent information and a heuristic search algorithm

Alejandro Estaña[1,2], Malik Ghallab[1], Pau Bernadó[2] and Juan Cortés[1]

[1] LAAS-CNRS, Université de Toulouse, CNRS, Toulouse, France

[2] Centre de Biochimie Structurale. INSERM, CNRS, Université de Montpellier, France

S1 - Scalability analysis

We applied the proposed method to five other proteins with increasing size, from 20 to 67 amino-acid residues: Trp-cage, WW-domain, BBL, CENP-B, and Villin (whose respective PDB IDs are: 1L2Y, 1QQV, 1E0M, 2WXC, and 1BW6). The folded states of the proteins are represented in Figure S1. The HDFS algorithm was applied to find paths between a fully-extended all-trans conformation and the folded state of each of these proteins. These experiments were not aimed to provide insights into the folding mechanisms of these proteins, but only to analyze the scalability of our method.

The performance indicators of the algorithm are summarized in Table S1, which gives the average values over 5 runs for these additional five proteins, as well as the values presented in the paper for Chignolin and DS119. We denote n the length of the protein, t the time it takes to find a path, m the number of states explored by the search, and d the depth of the search, i.e., the length of the found path. The analysis of our result can be summarized in the following points:

- As expected for a local propagation mechanism, the complexity of each search step is not a function of n. This is clearly shown by the ratio t/m which does not increase with n; its average is about 0.94 ms per search step. In comparison, each simulation step with usual MD approaches has a complexity in $O(n^2)$.
- As a backtrack search algorithm, HDFS is exponential with respect to d, the depth of the search, but not with respect to n, the size of the protein. The number of search states m grows as $m = d^b$, where b is the *branching factor*. Thanks to the heuristics guidance of the search, in our case b is very small, in average $b \simeq 1.03$. Again, b is not a function of n (we even observe smaller values of b for the larger proteins than for the smaller ones), but d grows with n.
- The overall complexity, in time or in the number of steps, increases with n, but with a quite reasonable polynomial growth, as illustrated with the three parameters α_1, α_2, and α_3 in Table 1. Their average values provide the following approximate growth: $t = n^{1.3}$, $m = n^{3.4}$, or $m = K \times n^{1.4}$, for K=1000 (this last function is more adequate given the constant value of t/m of about 1s for 1000 states). Note again that a simulation with MD would involve a number of steps growing with d (hence indirectly with n), each step being quadratic in n.

- As for any heuristics search algorithm, the performance figures are not smooth. Much more data would be needed to support precise average complexity models. However the above results give the main trends for the scalability of the approach: a quasi-constant t/m, a very small branching factor b, and a reasonable polynomial growth of the global complexity in the size of the protein. Clearly, the approach is scalable: for the largest system in our test set, Villin (with $n = 67$), the search algorithm explores about 4.5×10^6 states, requiring 35' of a single core standard processor. In contrast, results reported in the literature (Lindorff-Larsen et al., Science, 2011; reference [8] in the manuscript) indicate that in order to find folding pathways for a fast-folding protein such as Villin, MD simulations would require in the order of 10^9 steps, each of which being of quadratic complexity in n.

The results also show that the performance of the method depends on the structural elements in the protein. This can be clearly illustrated with the WW-domain. The folded structure of this protein is mainly composed of β-sheets, whereas the other four proteins mainly involve α-helices. Since the backbone of proteins has a natural propensity to twist, helical fragments are much more frequent that extended fragments, which lead to the formation of β-sheets. This explains the lower density of the states along the folding pathway for the WW-domain compared with the other four proteins. On the other hand, the presence of β-sheets in the folded structure significantly facilitates the search of folding paths form extended conformations, since these structural elements already correspond to extended fragments. This explains why the algorithm is faster on the WW-domain compared with the other proteins.

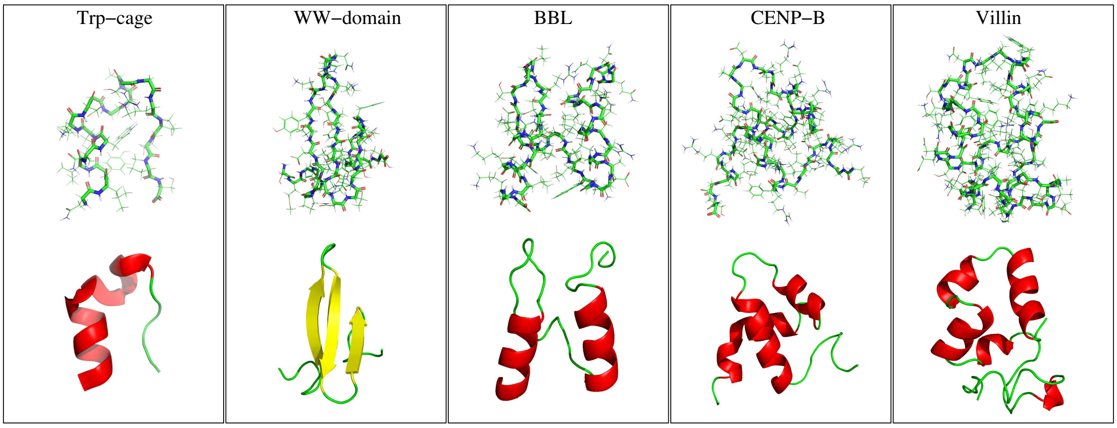

Figure S1: Structural representation of five proteins with increasing size used to analyze the scalability of the algorithm (in addition to Chignolin and DS119). The images at the top are detailed representations, in which thinker lines correspond to the protein backbone and thinner lines are used for the side-chains. The images at the bottom are "cartoon" representations that highlight the main structural elements: α-helices in red, and β-sheets in yellow.

	Chignolin	Trp-cage	DS119	WW-domain	BBL	CENP-B	Villin
$n =$ Nb residues	**10**	**20**	**36**	**37**	**47**	**56**	**67**
$t =$ CPU time (s)	**7.5**	**158.3**	**25.2**	**8.9**	**735**	**1096.5**	**2182.3**
$m =$ # states ($\times 10^3$)	3.1	235.1	70.6	30.6	1730.8	549.3	4507.7
$d =$ Path length (# steps)	96	508.0	158	165.5	2024.8	5041.6	3241.3
# backtracks ($\times 10^3$)	0.1	3.8	8.2	0.3	15.4	1.9	26.5
Path distance (rad)	7.3	16.0	11.3	9.4	33.3	139.7	45.7
Path density	18.5	39.0	124.4	6.3	147.9	52.4	45.8
t/m (in ms)	2.42	0.67	0.36	0.29	0.42	1.99	0.48
b	1.08	1.024	1.07	1.06	1.007	1.002	1.004
α_1	0.87	1.69	0.90	0.60	1.71	1.73	1.83
α_2	3.49	4.13	3.11	2.86	3.73	3.28	3.64
α_3	0.49	1.82	1.19	0.94	1.93	1.56	2.0

Table S1: Performance indicators of the HDFS algorithm on seven proteins with increasing size. CPU time was measured on an Intel® Core™ i7 processor at 2.8 GHz, using a single core. The last four parameters are defined as follow: $b = e^{(\log m)/d}$, $\alpha_1 = \log t / \log n$, $\alpha_2 = \log m / \log n$, and $\alpha_3 = (\log m - \log K)/\log n$. The average values are $t/m = 0.95$, $b = 1.03$, $\alpha_1 = 1.34$, $\alpha_2 = 3.46$, and $\alpha_3 = 1.42$.

S2 - Neighbor-dependent structural preferences

The distribution of the ϕ-ψ angles for a residue does not depend only on the nature of the neighboring residues, but also on their structure. This is illustrated for tripeptide Ser-Arg-Ala in Figure S2. One can clearly observe that when the ϕ-ψ angles of the Ser and Ala residues are constrained to be in the α region, the central Arg residue has a high probability to be also in this region. The same happens for the β/polyproline-II region.

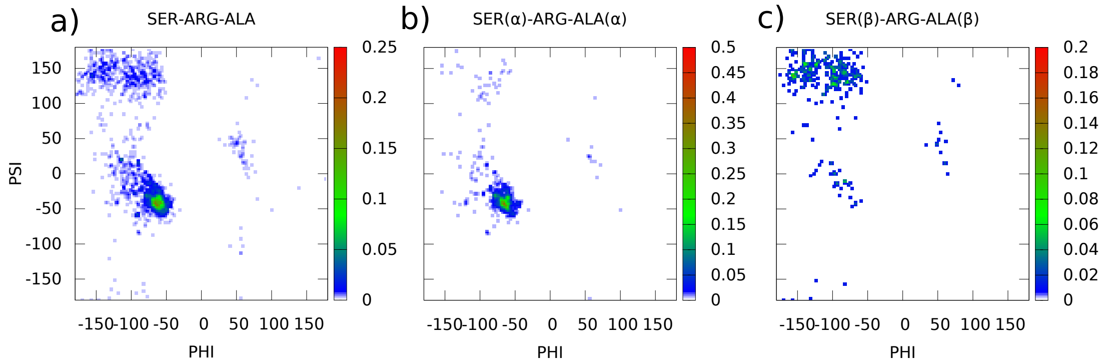

Figure S 2: Distributions of the ϕ-ψ angles of the central residue in a tripeptide, Ser-Arg-Ala, depending on the structure of the neighboring residues. (a) All the values for the central residue Arg, independently on the structure of Ser and Ala. (b) Values for Arg when Ser and Ala are in the α region. (c) Values for Arg when Ser and Ala are in the β/polyproline-II region.
